# Ultrasound- guided microwave ablation for symptomatic breast cysts: early outcomes and clinical implications

**DOI:** 10.3389/fsurg.2025.1544662

**Published:** 2025-08-22

**Authors:** Khanh Quang Huynh, Phat Thanh Nguyen, Khoi Van Nguyen

**Affiliations:** ^1^ Breast Unit, Cho Ray Hospital, Ho Chi Minh City, Vietnam; ^2^ Department of Thoracic and Cardiovascular Surgery, Faculty of Medicine, University of Medicine and Pharmacy at Ho Chi Minh City, Ho Chi Minh City, Vietnam

**Keywords:** breast cyst, microwave ablation, MWA, minimally invasive therapy, ultrasound guidance

## Abstract

**Background:**

Breast cysts are more common in premenopausal women (61.5%), particularly between ages 35–50. Microwave ablation (MWA) has shown advantages in treating symptomatic cysts.

**Objective:**

To evaluate early outcomes of ultrasound-guided MWA for breast cysts at the Breast Unit of Cho Ray Hospital.

**Methods:**

A retrospective review was conducted on patients who underwent MWA from August 2018 to March 2020.

**Results:**

Fifty-eight patients (mean age: 45.3) were included. Simple, complicated, and complex cysts accounted for 80.0%, 16.9%, and 3.1%, respectively. The mean cyst diameter was 23.86 mm. A size reduction exceeding 90% was observed in the ablated cyst at the 6 months follow- up, with the exception of 1 cyst with mural calcification that showed no response.

**Conclusion:**

MWA is a promising and safe treatment for symptomatic breast cysts, offering high success and low complication rates. Cyst mural calcification may affect treatment response and should be carefully assessed.

## 1 Introduction

Breast disease is divided into 2 main groups: benign and malignant, at the rates of 87.7% and 12.3%, respectively. This rate varies depending on age group (1). In the group of benign breast disease, cysts and fibrocystic changes are the two predominant groups.

Breast cysts are characterized as discrete, typically round or oval, fluid-filled lesions that originate within the terminal duct lobular units (TDLUs) of the breast parenchyma. Their pathogenesis stems from the obstruction of the distal ductules within these units, leading to their dilation and the accumulation of intraluminal fluid, ultimately forming the cyst. These cysts are commonly found in the upper half of the mammary gland and are more common in premenopausal women (65.1%) than in postmenopausal women (39.4%), with peak incidence between ages 35 and 50 (2). In the case of hormone replacement therapy, mammary cysts are most likely to appear in the postmenopausal period (3). There are three types of breast cysts: simple cysts, complicated cysts, and complex cysts (4).

Monitoring is required for simple and complicated cysts that caused no symptoms and evaluated by BIRADs 3 on ultrasound. Treatment is required for cysts that cause pain, breast tenderness, skin redness, or abscess to resolve symptoms. Fine Needle Aspiration (FNA) is a commonly utilized initial management strategy for symptomatic breast cysts, as well as for complex cystic lesions exhibiting a high risk of malignancy based on established scoring systems. However, a significant recurrence rate, reported to be approximately 80% (5), is a notable limitation. Surgical excision is generally indicated in cases of large or repeatedly recurrent cysts, as well as for cystic lesions with suspicious clinical or imaging characteristics despite cytologically benign aspirates. Nevertheless, the surgical procedure is associated with postoperative pain and potential cosmetic sequelae. Cochrane et al. (2003) has shown that patients are satisfied with their appearance if the estimated percentage of breast volume excised is less than 10% (6). Microwave ablation (MWA) offers many advantages: reducing treatment time and costs, reducing the length of stay, and less heat-sink effect than other thermal ablation methods (7). Microwave ablation is also applied for malignant lesions treatment (8), showing the safety and effectiveness in the treatment of benign breast lesions, which mainly is fibroadenomas.

Benign breast cystic disease often receives limited therapeutic intervention in the absence of patient-reported discomfort arising from symptoms and recurrence. Aspiration alone does not address the cause of cyst formation, leading to a high rate of recurrence. Among the less invasive treatments, microwave ablation has been shown to be effective in treating various organs.

This retrospective analysis examined breast cyst intervention cases managed at the Breast Unit of Cho Ray Hospital. Outcomes were evaluated based on diagnoses, performed procedures, and documented results throughout treatment and follow-up.

This study aimed to evaluate the safety and effectiveness of ultrasound-guided microwave ablation treatment of cystic breast disease.

## 2 Research objectives and methods

### 2.1 Research designs

Descriptive retrospective series of cases.

### 2.2 Research subjects

Data for this study were extracted from 58 medical records of patients who underwent microwave ablation for breast cysts between August 2018 to March 2020 at the Breast Unit, Cho Ray Hospital, Ho Chi Minh City.

### 2.3 Sample selection criteria

A case was considered as a sample if it met these following conditions: (i) Patient was diagnosed with breast cyst; (ii) Symptomatic mammary cysts did not respond to medical treatment and recurred after fine-needle aspiration; (iii) Negative cytology or pathology results; (iv) Microwave ablation treatment was applied.

### 2.4 Exclusion criteria

Patients were exclude due to the presence of other associate lesions that required biopsy for diagnostic evaluation.

### 2.5 Research variables

The characteristic variables of samples in this study included: age, gender, smoking habit, hypertension, coronary heart disease, diabetes, lung disease, kidney disease, cerebrovascular disease, and history of using endocrine.

Intervention-related variables included: Indication of intervention, type of interventional cyst, duration of intervention.

Outcomes-related variables included: Success rate of ablation procedure right after treatment, rate of size reduction during follow-up, and complications of pain, bleeding, infection, skin burns.

### 2.6 Measurement and statistical methods

Cyst size was measured based on maximum diameter due to the retrospective nature of the dataset and lack of volumetric data.

Data were collected and processed by using SPSS 25.

Qualitative variables are presented as frequencies and percentages.

Quantitative variables are presented as mean and standard deviation.

Qualitative variables were tested for correlation by chi-square test and Fisher's exact test, quantitative variables were tested for correlation by t-student test, ANOVA. *P* ≤ 0.05 was statistically significant.

### 2.7 Medical ethics

Microwave ablation procedure has been approved by the scientific council of Cho Ray Hospital.

## 3 Results

From August 2018 to March 2020, 58 patients with a total of 209 cysts were found via ultrasound at the Breast Unit of Cho Ray Hospital. In most cases, cysts are located at the upper and outer of the breast, the rate of polycystic is up to 84.5%. The average size of cysts detected by ultrasound is 14.2 mm. Fine-needle aspiration is performed for symptomatic cysts, as well as complicated and complex cysts which have a high risk of malignancy. A total of 65 benign cysts showed no response to aspiration, medical and hormonal treatment. Microwave ablation was indicated for these cysts. Among the 209 cysts identified in 58 patients, only 65 cysts were treated with microwave ablation based on the following criteria: no response to aspiration, benign cytology or pathology results, and failure of medical therapy. A total of 144 cysts were excluded from the study cohort due to predefined sample selection criteria detailed in Section 2. These excluded cases fell into two main categories: small (less than 10 mm) and asymptomatic cysts—where the size restriction was specifically implemented to ensure accurate targeting and safe ablation, consistent with findings by Yang et al. (9)—and larger cysts that showed a positive response to medical, hormonal, or aspiration treatments.

Characteristics of 65 benign cysts in 58 patients are provided by ultrasound scanning.We utilized the ultrasound equipment available at the Breast Unit of Cho Ray Hospital. Due to limitations in the available device functionality, the assessment and classification of breast cysts were performed through conventional 2D and color Doppler modes, without the application of elastography or contrast-enhanced ultrasound (CEUS). The average size of the microwave ablation cyst was 23.86 mm, with the range extending from 10 mm to 56 mm (Table 1). Numbers and size ratio of ablation cysts are detailed in Table 2. Categorization of the cysts revealed the following distribution: simple cysts constituted 80% of the sample, complicated cysts 16.9% and complex cysts 3,1%.

**Table 1:** Diameters of the breast cysts were performed with microwave ablation (*n* = 65).

Max.	Min.	Average
56 mm	10 mm	23.9 ± 11.4 mm

The treated cysts exhibited a substantial size.

**Table 2:** Size distribution of breast cysts treated with MWA (*n* = 65).

Size	Quantity (*n* = 65)	Percentage (%)
d < 20 mm	24	36.9
20–30 mm	29	44.6
d > 30 mm	12	18.5

The majority of treated cysts presented diameters ranging from 20 to 30 mm.

Sample characteristics in this study had an average age of 45.3. Among 100% female patients, the rates of premenopausal and menopausal women are 61.5% and 39.4%, respectively. Palpable cysts were found in 91.4% patients, and pain presented in 42.4% of total cases. All patients were treated with fine needle aspiration and were found to be unresponsive. Before being prescribed microwave ablation, 32.7% of patients had received medical treatment. The proportion of patients using hormone is 10.3%. The mean time from diagnosis until receiving microwave intervention was 19.7 months.

Microwave ablation was performed under ultrasound guidance. Antenna sizes are chosen depending on lesion sizes. To ablate small cysts (d < 2 cm), mini antennas (16G, 1 cm) were used, while small antennas (16G, 2 cm) were chosen for cyst larger than 2 cm in diameter. The temperature was set at 70°C, and local anesthesia was applied for every case. Treatment time is about 2.5 min for simple and complicated cysts, and 3.25 min for complex cysts. Results shown that MWA is safe and highly effective, without major complications.

All lesions (*n* = 65) underwent microwave ablation. At the 6-month follow-up, the incidence of palpable lesions decreased significantly from 91.4% to 5.2%. Furthermore, a 50% reduction was observed in the number of patients reporting pain symptoms. These preliminary findings suggest the efficacy of microwave ablation in treating these lesions. Table 3 details the number of patients presenting with palpable lesions prior to and following microwave ablation.

**Table 3:** Patients with palpable lesions before and after microwave ablation (*N* = 58).

Tactile Lesion classification	Before		After	
	Q'ty	%	Q'ty	%
Palpable	53	91.4	3	5.2
Non-palpable	5	9.6	55	94.8

A 94.3% reduction was observed in the number of patient with this clinical sign.

Radiologically, the size of cysts was significantly reduced after 1, 3 and 6 months of follow-up. Average reduction rate reached 99.5%, 92.2% and 91.5% for simple, complicated, and complex cysts, respectively, after 6 months (Table 4). During post- MWA monitoring, no case of recurrence after microwave treatment was recorded.

**Table 4:** Treatment response rate across different cyst groups over a 6-month observation period.

Cyst groups	Reduction rate (%)		
	After 1 month	After 3 months	After 6 months
Simple cysts	96.4 ± 1.4	98.1 ± 0.9	99.5 ± 0.5
Complicated cysts	85.9 ± 4.2	88.9 ± 4.4	92.2 ± 4.8
Complex cysts	81.0 ± 1.4	86.0 ± 1.4	91.50 ± 2.1

A gradual decrease in cyst size was observed with the simple cyst group displaying the most substantial reduction.

## 4 Discussion

In our study, the prevalence of breast pain was 42.4%, with no statistically significant difference observed in the occurrence of this symptom between menstruating and postmenopausal women (*p* > 0.05). While aligning with Morrow's observation that breast pain is a primary symptom prompting patients to seek medical consultation (10), our finding differs from his assertion that this is most common in premenopausal individuals.

The size of mammary gland cysts detected via ultrasound in our study, with a mean of 14.2 mm (range: 3–56 mm), aligns with findings from Taskin and Tez (11, 12). Specifically, Taskin et al. reported a mean diameter of 16 mm (range: 4–42 mm) in their investigation of complex cysts (11). Similarly, Tez et al.'s study on 246 non-palpable complex cysts documented a mean size of 15 mm, with a range of 4–41 mm (12).

In comparison to the classification results in Berg et al. (13), our study demonstrated a similar ratio of simple cysts, but it revealed a reciprocal relationship in the proportion of complicated and complex cysts. Specifically, the incidence of complicated cysts in our study was half that reported by Berg et al., while the rate of complex cysts was twice as high. This difference may be attributable to the variation in sample size and the racial characteristics of the study population. Chang et al. (14) employed a similar sampling method to this study, focusing on symptomatic cysts that were confirmed as benign by cytology or pathology. However, their classification method differed, with the inclusion of three additional categories: clusters of cysts, cysts with thin walls, and cysts where solid components constituted less than 50% of the volume. These divergent classification criteria likely contributed to the dissimilarities in outcomes. Furthermore, variations in sampling methods may also account for the difference in classification rates. As exemplified by Buchberger et al. (15), their focus was on asymptomatic breast cysts detected via ultrasound, contrasting with the symptomatic focus of our study.

The selection of instrumentation and the microwave ablation (MWA) treatment protocol adhered to the methodology detailed in Section 3. MWA was subsequently applied to 65 benign cystic lesions across a cohort of 58 patients. Our procedural approach exhibited minor deviations from the techniques described by Yu et al. (16) and Zhang et al. (17), primarily concerning the modulation of temperature vs. power parameters. In the study conducted by Yu and colleagues, 198 lesions in 122 patients underwent microwave ablation with a mean power output of 28.3 ± 6.2 W and an average ablation duration of approximately 3.2 min. Furthermore, they applied a water dissection technique to separate lesions from adjacent tissues, including the skin, mammary gland, and areola. The reported outcomes indicated complete ablation in 99.5% of cases, with only a single large lesion (3.6 cm) being partially removed. Similarly, Zhang et al. (17) utilized MWA to treat 205 benign breast lesions in 182 patients, employing a consistent power output of 40 W across all cases. Post-procedural contrast-enhanced ultrasound evaluation revealed complete resection in 97.8% of the treated lesions.

**Figure 1:**
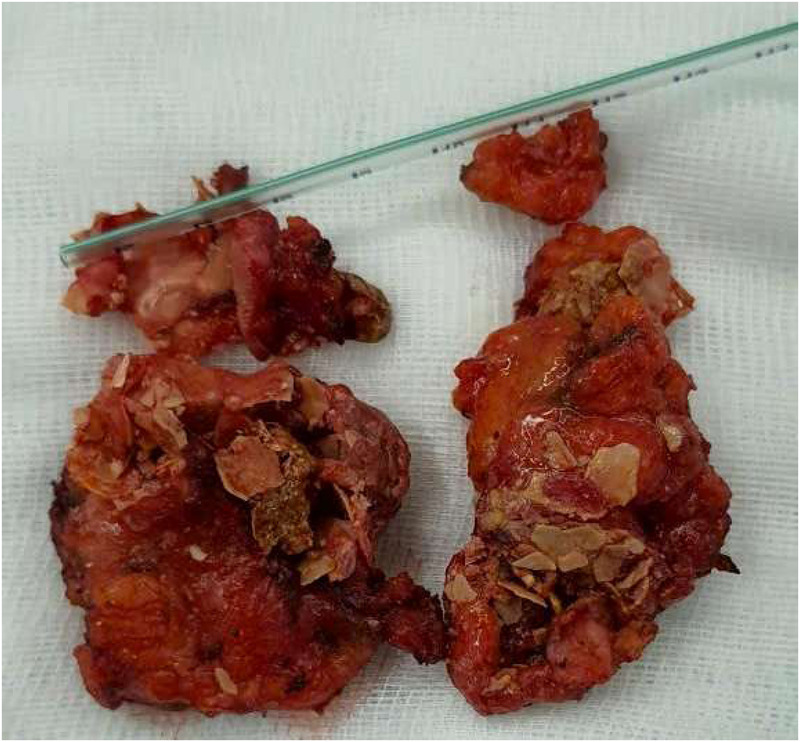
Circumferential mural calcification in a breast cyst, demonstrating unresponsiveness to MWA due to the rigid wall impeding resorption.

Among the three palpable lesions, one was determined as a complicated cyst (4) exhibiting a calcified wall and showing no significant size reduction during six months of follow-up. However, open surgical excision revealed complete necrosis of the internal tissue, attributed to microwave ablation. The circumferential calcified cyst wall likely acted as a physical barrier, preventing phagocytic activity (18, 19), which resulted in no observable change in lesion size despite complete ablation of the internal content (Figure 1). The remaining two palpable lesions were determined to be the post-ablation fibrosis, causing architectural distortion and rearrangement of physiological tissue organization, a phenomenon frequently associated with increased breast stiffness pathology, as reported by Prokop et al. (20).

Regarding patient-reported outcomes, the incidence of breast pain post-intervention decreased from 42.4% to 20.7%. Our assessment suggests this pain was primarily attributable to the effects of microwave ablation rather than residual pain from the breast cysts themselves. This statistically significant reduction in pain (*p* = 0.000125) indicates that intervention for symptomatic breast cysts can provide pain relief. Furthermore, this minimally invasive method obviates the need for surgical or minor surgical cyst removal.

In our study, no cases required MWA re-intervention. However, there were 6 cysts (9.2%) that needed aspiration to increase the effectiveness of treatment and 01 cyst (1.5%) with mural calcification required open surgery excision. These cases all into complicated cysts and complex cysts.

During the intervention, no instances of skin burns were recorded. Skin burns in this context can arise from two primary mechanisms. The first is thermal conduction along the antenna, which can elevate the temperature at the skin puncture site. Heat generated at the needle tip is transferred proximally along the shaft, potentially causing skin burns. To mitigate this risk, maintaining the ablation temperature at 70°C is advisable. The second mechanism is the proximity of lesions to the skin surface. When lesions are superficially located, the thermal energy applied can readily diffuse to the skin and adjacent tissues, increasing the susceptibility to burns. To address this, the injection of an anesthetic or saline solution to create a separation between the lesion and the skin surface is recommended. This technique has demonstrated its efficacy in the study by Xu et al. (21), where the perilesional injection of distilled water during ablation procedures yielded comparable outcomes to our observations regarding the absence of skin burns. Referring to another study on microwave ablation by Zhang et al. (17), skin burns were recorded at a minimal rate of 0.1%. This is significantly lower compared with the higher rates of 10% and 26.7% observed in laser cauterization of fibroadenomas (21, 22). Furthermore, the integration of ultrasound guidance in MWA has been associated with a significant reduction in major complications, as indicated by (23), suggesting that real-time imaging plays a crucial role in enhanced procedural safety.

The difficulty of follow-up is that after intervention, benign cysts may be recognized as a high BIRAD lesion on ultrasound scanning and thus lead to unnecessary intervention.

Table 4 presents the lesion dimensions at 1, 3, and 6 months post-ablation, demonstrating a mean reduction rate of 89.3% ± 12.7%. This observation indicates a statistically significant decrease in cyst volume following microwave ablation (MWA). Consistent with the findings reported by Zhang et al. (17), breast cysts exhibited a greater degree of volumetric reduction in response to this intervention when compared to benign solid tumors.

Patients underwent periodic follow-up evaluation from 3 to 12 months, contingent upon the response of their post-ablation lesions. The mean follow-up time was 8 months. In our study, palpable lesions demonstrated a significant reduction in number, from 53 (81.15%) to 4 (6.3%) cases after treatment and follow-up. Comparatively, a study by Yu et al. (16) also reported a decrease in the rate of palpable lesions after 14 months, albeit with a substantially higher residual rate (from 90.2% to 45.9%).

The results of ultrasound examination at six months post-ablation revealed a 98.5% complete response rate in cystic lesions. This finding exhibited a strong similarity to the results obtained by Zhang et al. (17). With respect to cosmetic outcomes, a 100% patient satisfaction rate was documented after the procedure. Additionally, the study noted the absence of adverse events, lesion recurrence, or instances of initially benign lesions being classified as malignant.

It is crucial to acknowledge that, as previously mentioned, the application of microwave ablation can induce alterations in tissue architecture. These modifications may subsequently lead to the mischaracterization of post-interventional benign lesions as high BI-RADS categories under ultrasound, potentially resulting in unwanted interventions. Consequently, meticulous monitoring of patients’ clinical history is imperative during breast cancer screening programs and subsequent related breast examinations to facilitate accurate assessment and avoid unnecessary procedures.

## 5 Conclusion

Microwave ablation represents a viable and safe treatment for breast cysts, demonstrating high efficacy and favorable aesthetic outcomes with minimal postoperative complications. For patients with breast cysts refractory to medical therapy and fine-needle aspiration, microwave intervention constitutes an effective therapeutic alternative that should be considered. Limitations of this study include a small sample size and a short follow-up period. Future prospective studies with larger and more diverse populations, extended follow-up durations, and standardized procedural protocols are needed to further confirm the therapeutic benefits of ultrasound-guided MWA for breast cysts. Given the restricted and non-representative sample of calcified cysts in this study, definitive conclusions regarding their response cannot be drawn. Further research should incorporate a wider assessment of MWA's efficacy in treating calcified cysts, particularly those with mural calcification.

## Data Availability

The original contributions presented in the study are included in the article/Supplementary Material, further inquiries can be directed to the corresponding authors.
